# Decreased Retinal Thickness in Type 1 Diabetic Children with Signs of Nonproliferative Diabetic Retinopathy

**DOI:** 10.1155/2018/1078531

**Published:** 2018-04-26

**Authors:** P. Ruiz-Ocaña, P. Espinoza Requena, A. Alonso-Ojembarrena, P. Alemany Márquez, S. Jiménez Carmona, A. M. Lechuga-Sancho

**Affiliations:** ^1^Diabetes and Metabolism Unit, Department of Pediatrics, University Hospital Puerta del Mar, Cádiz, Spain; ^2^Department of Ophthalmology, University Hospital Puerta del Mar, Cádiz, Spain; ^3^Neonatology Unit, Department of Pediatrics, University Hospital Puerta del Mar, Cádiz, Spain; ^4^Department of Surgery, School of Medicine, Cádiz University, Cádiz, Spain; ^5^Department of Mother and Child Health and Radiology, School of Medicine, Cádiz University, Cádiz, Spain

## Abstract

The retina functions as a neurovascular unit. How early vascular alterations affect neuronal layers remains controversial; early vascular failure could lead to edema increasing retinal thicknesses, but alternatively neuronal loss could lead to reduced retinal thickness. *Objective*. To evaluate retinal thickness in a cohort of pediatric patients with type 1 diabetes mellitus (PwT1DM) and to analyze differences according to the presence or absence of nonproliferative diabetic retinopathy (NPDR), poor metabolic control, and diabetes duration. *Patients and Methods*. We performed retinographies and optical coherence tomography (OCT) (TOPCON 3D1000®) to PwT1DM followed at our center and healthy controls. Measurements of the control group served to calculate reference values. *Results*. 59 PwT1DM (age 12.51 ± 2.59) and 22 healthy controls (age 10.66 ± 2.51) volunteered. Only two PwT1DM, both adolescents with poor metabolic control, presented NPRD. Both showed decreased thicknesses and retinal volumes. The odds ratio of having decreased retinal thickness when signs of NPDR were present was 11.72 (95% IC 1.16–118.28; *p* = 0.036). *Conclusions*. PwT1DM with NPDR have increased odds of decreased retinal thicknesses and volumes. Whether these changes are reversible by improving metabolic control or not remains to be elucidated.

## 1. Introduction

Diabetic retinopathy (DR) is one of the microvascular complications associated with T1DM, being the most frequent cause of blindness in “active population” (adults between the ages of 20 and 74) [1]. It is estimated that one-third of people with diabetes have signs of retinopathy, one-third of whom suffer from vision-threatening diabetic retinopathy (VTDR, defined by the presence of proliferative retinopathy and/or macular edema), with approximately 93 million people in the world with DR, 17 million with proliferative DR, 21 million with diabetic macular edema, and 28 million with VTDR worldwide [2].

The mechanisms involving DR development include the following: hyperglycaemia may cause sorbitol accumulation in retinal cells, increased polyol metabolism, advanced glycosylation end product (AGE) elevation in the extracellular fluid, increased protein kinase C and hexosamine pathway activity [3], upregulation of growth factors and proinflammatory cytokines, hyperactivation of the renin-angiotensin system, and exacerbated production of superoxides. The mechanism by which these cytokines contribute to vascular and neuronal apoptosis is not yet clear and may respond to excitotoxicity, oxidative stress, and/or mitochondrial dysfunction [3].

In preclinical retinopathy, no ophthalmoscopic alterations are observed, although alterations of the neurovascular unit have already been demonstrated at this point. The neurovascular unit is a histological concept explaining that, under normal conditions, endothelial cells and pericytes, astrocytes, Müller cells, and neurons are closely related and connected, establishing the so-called blood-retinal barrier, in order to favor an adequate flow of nutrients, an ionic environment appropriate for neurological signaling, synaptic transmission, and adaptive responses that allow an adequate visual function [4].

Thus, the damage induced by DR would not be limited to an isolated angiopathy but would involve both the vascular and neuronal compartments. This statement is based on the demonstration of early subtle changes in microvascular hemodynamics [5], neuronal functionality in studies performed by electroretinography [6, 7], and the decrease in thicknesses of the nerve fiber layer of the retina [8] in these early preclinical stages of retinal involvement.

In children, although it is common to demonstrate ophthalmoscopic findings such as isolated retinal microaneurysms or small unilateral hemorrhages (all of which are signs of nonproliferative diabetic retinopathy (NPDR)), it is extremely rare to find preproliferative retinopathy, proliferative retinopathy, or macular edema, regardless of the degree of metabolic control or the time of evolution of diabetes [4], with a clear tendency in our days to the decrease in the detection of retinopathy [4, 9, 10].

To date, the younger age at which severe DR (preproliferative or proliferative) has been documented is 15 years of age, and the shortest documented duration of diabetes since its clinical onset to severe DR presentation is 5 years [11]. Besides diabetes duration and patient's age, another independent risk factor for DR development is the age at diabetes onset; those who were under 5 years of age at the start of their clinical diabetes have decreased risk of DR development [12].

Since its introduction in clinical practice, optical coherence tomography (OCT) has been used in research on diabetic retinopathy, given its ability to accurately determine retinal thickness, being a noninvasive technique. OCT enables the objective and quantitative assessment of retinal volumes and thicknesses, having excellent reproducibility in children with respect to adults [13, 14]. The main problem in the realization of OCT in children is the interpretation of the measurements when the devices lack a specific reference database in the pediatric age.

Different reference values for macular and papillary structures have been published, rendering great variability of the measurements depending on the device used (up to 26 *μ*m in the same patient). Thus, investigators and clinicians need to calculate their own reference values for the OCT device used, since different software calculate their measurements based on different delimitations in the external segmentation line [15–19].

Previous publications have found retinal thickness alterations in both directions: on the one hand, there are reports of increased retinal thickness, both in established diabetic retinopathy [20] and in clinically significant diabetic macular edema [21–23], pointing at OCT's suitability as a screening method for the subclinical macular edema in diabetic patients [24]. On the other hand, decreased thicknesses have also been reported [8, 25], suggesting neuronal cell loss as the cause. Finally, there are also a number of studies that do not appreciate significant differences in any way [26, 27].

## 2. Objective

The aim of this study is to compare retinal thickness in pediatric patients with type 1 diabetes mellitus (PwT1DM) diagnosed with nonproliferative diabetic retinopathy (NPDR) with those obtained in healthy subjects.

## 3. Patients and Methods

We performed a transversal comparative study between the cohort of PwT1DM followed at the Children's Diabetes Unit of Puerta del Mar University Hospital (Cadiz) and healthy children and adolescents aged between 4 and 18 years. The study protocol was evaluated and accepted by our institution's ethics committee for clinical research, and written informed consent was obtained from all study subjects and/or their legal guardians.

Patients were recruited from our Diabetes Unit, and controls were recruited from patients' close relatives or friends, aiming at similar age and sex distribution in both groups. Clinical variables involving demographics, anthropometry, characteristics of the diabetic process, and degree of metabolic control were registered from their electronic medical records after informed consent was obtained. As a variable of diabetes control, the glycated hemoglobin levels, its average levels in the year before, and the mean in the whole “historical period of illness” were registered at the time of ocular exploration. In addition, the variation coefficient and standard deviation of the average levels of the historical period were determined as measures of the dispersion of these levels.

The analytical determinations were carried out by the usual methods (colorimetry and ECLIA) in analytical platforms C-711 and E-170 of Roche Diagnostics in the Clinical Analysis Service of our center.

**Figure 1:**
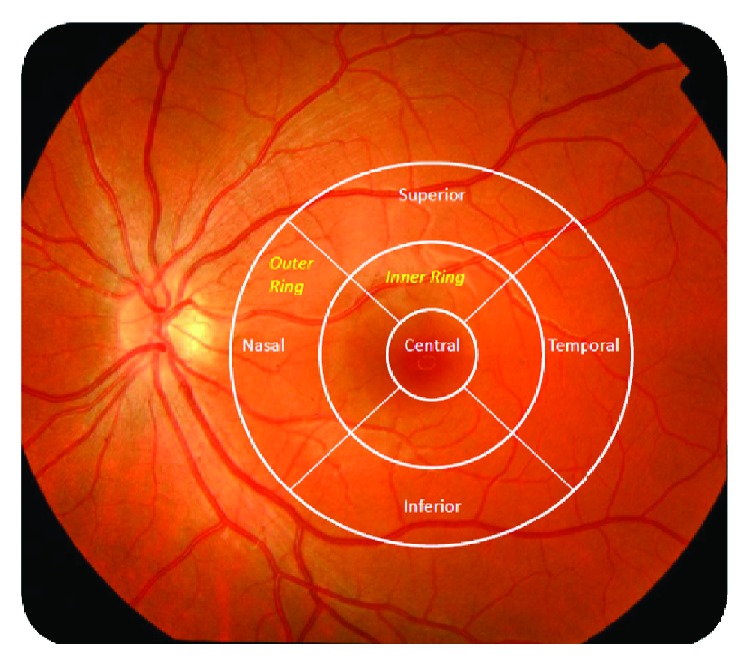
Outline of the macular regions according to the ETDRS's grid.

The ophthalmological evaluation consisted of anterior chamber study, intraocular pressure, autorefractometry, and visual acuity. The retina was explored by conventional retinography and OCT, using the 3D OCT-1000 spectral domain device (TOPCON, Japan) for macular and papillary study. The study map used was the grid proposed by the Early Treatment Diabetic Retinopathy Study (ETDRS), composed of three concentric circles of 1, 3, and 6 mm in diameter corresponding to the fovea, inner ring, and outer ring, respectively. Each ring is subdivided into superior, nasal, inferior, and temporal depending on its location with respect to the midline as shown in Figure 1.

### 3.1. Exclusion Criteria

Patients with other types of diabetes different from type 1 were not included, as well as patients with less than 12 months from diabetes clinical onset at the time of the study. Those presenting limitations for undergoing the exploration or for the interpretation of the results, such as subjects unable to collaborate adequately to complete the ocular explorations, those presenting a refractive error > 5.5 diopters (D) or astigmatism greater than 3 D, those with a visual acuity (VA, Snellen Visual Acuity Scale) of less than 0.7 with a difference of vision greater than one line of the optotype between the AV of both eyes, and those with intraocular pressure (IOP) greater than 21 mmHg or those presenting other conditions, such as strabismus, amblyopia, or the presence of pathology that may cause changes in retinal thickness such as chorioretinal scars, myelin fibers, or papillary druses, were also excluded.

Finally, we discarded those OCT reports with a signal strength lower than 41, since this strength is considered the reliability limit of the exploration.

We used the statistical packages IBM © PASW STATISTICS © (IBM Corporation, Somers, NY, USA) in its version 18 and GraphPad Prism 6.0 for the statistical analysis of the data. We express the qualitative variables as number and percentage and the quantitative variables as average and standard deviation. First, we performed a descriptive study and used the data from controls to calculate the reference values (in the form of thickness and volume percentiles) of each of the macular and papillary sectors of the grid. Subsequently, we calculated the same percentiles for the T1D patients and compared these with the controls' using Student's *t*-test for independent samples, previously evaluating the equality of variances with the Levene test and establishing a level of significance of 95%, determining the difference with a *p* value under 0.05 as significant.

For the variables expressed as a scale, a study of bivariate correlations was performed using Pearson coefficients in the study of parametric correlations and Spearman coefficients in the study of nonparametric correlations. It was determined as significant with a level less than or equal to 0.05 (bilateral), and it was considered relevant when presenting a value of *R* greater than or equal to 0.4.

Finally, we calculated the odds ratio of presenting decreased thicknesses as a function of having signs of NPDR or not, having greater or less than 5 years of diabetes duration, having a mean HbA1c level of greater or less than 8.5%, or having diabetic clinical onset presentation before or after the 5 years of age (with a CI of 95%) and tested for significance using the *Z* statistic.

## 4. Results

64 PwT1DM and 36 controls volunteered. In 3 PwT1DM and 9 controls, it was not possible to complete the different exploratory techniques due to poor collaboration. Two more PwT1DM and 5 more controls met the exclusion criteria after having undergone exploration (increased intraocular pressure, strabismus, and astigmatism) and were not included in the analysis. Finally, our sample consisted of 59 PwT1DM (age 12.51 ± 2.59 years, ranged from 7.24 to 16.93 years) and 22 healthy controls (age 10.66 ± 2.51 years, ranged from 6.41 to 16.17 years).

The clinical, demographical, and anthropometrical characteristics are summarized in Tables 1 and 2. As seen in Table 1, no relevant differences were found in any of the anthropometrical parameters between PwT1DM and controls. As shown in Table 2, our PwT1DM have a relatively young average age at diabetes clinical onset, and the mean duration of diabetes since the onset to the study is over 5 years. Most of our PwT1DM were under multiple insulin dose regimen with long-acting analogues as basal insulin and ultrafast analogues as rapid-acting insulin for boluses.

Our sample's degree of diabetes metabolic control is summarized in Table 3. The glycosylated hemoglobin (HbA1c) values were analyzed both at the time of exploration and during the previous year, as well as during the evolution of the disease from debut to examination.

After careful examination, 3 OCT reports were discarded for not meeting the minimum quality, 25 were considered unusable for being off-centered, 4 were excluded due to partial signal absence (blinks), and two more reports presented artifacts, and only the data from undisturbed sectors were taken into account in these two. Hence, our final total consisted of 139 OCT reports of appropriate quality. Discarding off-centered scans increases the internal validity of our data.

Since there are no reference values for retinal thicknesses and volumes in childhood with the device we used (TOPCON 3D OCT-1000), we proceeded to calculate our own for each macular and papillary sector measurement, using the data from the group of healthy controls (Table 4). From now on, we considered “decreased retinal thickness” values below the third percentile of the reference data and “increased retinal thickness” values over the 97th percentile.

When comparing both study groups' values, we found no differences in any thickness or volumes (Table 5).

In our group of PwT1DM, we found not even one with a retinographic image compatible with subclinical macular edema or vision-threatening retinopathy. In only two cases, we found pathological images suggestive of incipient diabetic retinopathy (IDR): one patient presented microaneurysm and the second patient flame hemorrhage. Thus, we had an IDR incidence of 3.3% of the sample. The patient with microaneurysm (case 1) was a 14.8-year-old girl, with nearly 10 years of diabetes duration, obesity, and poor metabolic control all throughout the disease, with a mean annual HbA1c of 9.1% and 8.4% in the year of the study point. The patient with flame hemorrhages (case 2) was a 13.4-year-old boy, with nearly 9 years of diabetes evolution and also poor metabolic control, with mean annual HbA1c levels of 9.1% since clinical onset and the same levels in the year and at the time of the study. None of these had hypertension, neither microalbuminuria.

When comparing the thicknesses of these two patients' retinas with the reference values, we found that both patients presented at least two values below the third percentile and at least 3 between the third and 10th percentiles (Table 6).

The power of our contrast to detect the difference in the superior inner thickness (the variable with the largest difference between controls and PwT1D with NPDR) as statistically significant is 93%, assuming an alpha error of 0.05 in a bilateral contrast with 40 subjects in the control group and 2 in the PwT1D + NPDR.

However, these were not the only patients in whom we found thicknesses or volumes below the third centile. We also identified 11 more PwT1DM without retinographic images suggestive of IDR, with at least one measurement below the third centile. Hence, we performed a multiple correlation study to try to identify those factors related to an increased risk of decreased thicknesses or volumes, but we did not appreciate significant correlation between any retinal measurement and the degree of metabolic control expressed in the levels of glycosylated hemoglobin or any of the other variables analyzed (age at diabetes onset, current age, diabetes duration, sex, pubertal stage, type of therapy, etc.) (data not shown).

We calculated the odds ratio of presenting decreased retinal thicknesses as a function of acceptable (HbA1c < 7.5%) or poor metabolic control (HbA1c > 8.5%) or as a function of the age at diabetes onset and found no significant odds (*p* = 0.15 and *p* = 0.36, resp.). The time of diabetes evolution > 5 years at the moment of the study did have an increased odds ratio of presenting decreased retinal thicknesses (OR 2.18), but it missed statistical significance (*p* = 0.09). However, the odds ratio of having decreased retinal thickness when signs of NPDR were present was 11.72 (95% IC 1.16–118.28; *p* = 0.036).

## 5. Discussion

The variations in retinal thickness in adult PwT1DM have been the subject of study in recent years, with controversial results. Different studies find an increase in macular thickness (global or sectoral) [22, 28], hypothesizing that the cause of this increase could respond to the accumulation of fluid between the layers of the retina secondary to the loss of BHR function and early and subclinical stage of diabetic macular edema. Others, however, find decreased thicknesses [8, 25] supporting the hypothesis of neuronal cell loss as the first event of diabetic retinopathy, prior to vascular damage. Finally, there are also a number of studies that do not appreciate significant differences in one way or another, stating that the time of disease evolution can play a decisive role in the findings and in relation to the pathogenic phenomena of the disease [26, 27].

Studies in children are more recent and similarly controversial; while some studies show a thickening of the retinal tissues in diabetic patients [27], others report the opposite [29]. In the literature, differences in subfoveal choroidal thickness between PwT1DM and healthy controls are not observed [27], neither in the retinal measurements nor in the fiber layer and nor in ganglion cells when comparing PwT1DM1 without diabetic retinopathy and healthy controls [26].

Before the Diabetes Control and Complications Trial (DCCT) of 1993 [30], a DR prevalence up to 41-42% in the United States [31] and Australia [32] and even 46% in some regions of Europe [33] had been reported in adolescents. The findings of the DCCT showed that intensive therapy in children between the ages of 13 and 17 years reduced the risk of developing DR up to 53% [30], and since then, this therapeutic option is the main one used in the pediatric age. Despite the difficulty of achieving the target HbA1c proposed as optimal (median HbA1c in 7.5%), different publications in recent years agree on the progressive decrease in the overall incidence of the onset of diabetic retinopathy (DR) in the general population and in the pediatric population in particular [34, 35].

When considered as a group, our PwT1DM did not present differences in thicknesses or volumes in any of the ETDRS sectors of the retina measured overall, but we found an incidence of 3.38% of incipient diabetic retinopathy. This incidence is lower than that previously reported by others. In 2011, Cho et al. reported, in a population similar to that of our study (adolescents between 11 and 17 years of age and a time of evolution of T1DM between 2 and 5 years), a decrease in the incidence of retinopathy from 16% (year 1990) to 7% (from 2002 onwards), with a metabolic control of the cohorts expressed in median of HbA1c with values of 8.7 and 8.2% [36]. Downie et al. also reported the data of different historical cohorts of adolescent PwT1DM from 1990 to 2009, with a DR incidence decreasing from 53% to 12%, with median HbA1c levels of 9.1% and 8.2, respectively [35].

Taking into account that the median HbA1c in our population at the date of exploration and in the previous year of exploration is below the values of these studies (Table 2), our data support the role of an improved metabolic control to decrease the incidence of DR in children.

The two cases with signs of IDR shared two risk factors associated with the development of retinopathy, such as poor metabolic control [37–42] (both had HbA1c levels over 9%) and the time of evolution of disease [43], around 10 years [11]. In addition, case 1 had a significant obesity, an independent risk factor for the onset of retinopathy [44].

The localization of the decreased thicknesses was not the same in both cases. In case 1, it affected mainly the inner ring, and in case 2, it was generalized. These observations are in accordance with those of the studies reporting decreased retinal thickness and volumes in patients with incipient diabetic retinopathy [8].

We found no relevant correlations between the different analytical, demographical, anthropometric, and ocular variables. These either were not significant or did not reach *R* values under 0.4. This lack of correlations could be striking although it is likely that a larger sample size could increase the power of the correlation study.

We cannot neglect commenting on other limitations of our study. The technology that we used did not facilitate the automated and individualized measurement of the different layers of the retina, which would have detected thickening or retinal thinning located in specific layers of the retina. In addition, the cross-sectional design of the study does not allow us to analyze the temporal, permanent, or evolutionary nature of our findings or to calculate relative risks. Since different studies have shown decreased thicknesses while others have reported increased thicknesses, it is plausible to hypothesize that such changes could be evolutionary over time, and the design of our study does not allow for this differentiation. The low incidence of IDR in our sample and of reduced retinal measurements also makes it difficult to find significant associations with clinical variables; thus, multicenter studies using the same technology, as well as prospective study designs, would be desirable for the future.

In summary, taking into account the mentioned limitations, in our work, we have not observed differences between the measurements of thickness and macular volumes between PwT1DM as a group and healthy controls; we have found a low incidence of incipient diabetic retinopathy in our sample and an increased odds ratio of reduced retinal thicknesses in PwT1DM with IDR. The time of diabetes evolution also tended to increase the odds of reduced retinal thickness, but it did not reach statistical significance, probably due to the limited sample size.

We believe that our data have mainly three implications; firstly, we cannot advocate for the use of retinal thickness measurements as a DR-screening tool in children and adolescents with relatively good metabolic control of their diabetes and no ophthalmoscopic changes. Secondly, OCT scans could be of value in the study of PwT1DM and signs of incipient diabetic retinopathy to better characterize retinal changes. Lastly, our data suggest that in order to identify which changes happen first in the development of DR, it would be advisable to include the study of different retinal layers, in a prospective manner, especially in those adolescents with poor metabolic control of their diabetes.

## 6. Conclusions

PwT1DM with no ophthalmoscopic changes suggesting DR do not present differences in retinal thicknesses or volumes when compared to healthy controls. Adolescent PwT1DM with NPDR have an increased odds ratio of presenting decreased retinal thicknesses and volumes. Whether these changes are reversible by improving metabolic control or not remains to be elucidated.

## Figures and Tables

**Table 1:** Anthropometric characteristics of the total population.

	Controls	PwT1DM
Subjects (*N*)	22	59
Decimal age (years)	10.66 ± 2.51	12.51 ± 2.58
Height (*Z*-score)	−0.46 ± 1.12	−0.12 ± 1.09
Weight (*Z*-score)	−0.28 ± 1.08	−0.14 ± 0.89
Body mass index (kg/m^2^)	18.57 ± 4.11	19.63 ± 3.37
Body mass index (*Z*-score)	−0.05 ± 0.99	−0.09 ± 0.79
Waterlow index (%)	96.604 ± 15.88	98.353 ± 14.65
Systolic blood pressure (mmHg)	110.26 ± 14.24	111.17 ± 10.32
Systolic blood pressure (*Z*-score)	0.07 ± 1.12	0.40 ± 0.98
Diastolic blood pressure (mmHg)	61.83 ± 7.74	63.40 ± 7.90
Diastolic blood pressure (*Z*-score)	0.12 ± 0.62	0.07 ± 0.669

**Table 2:** Clinical characteristics of the PwT1DM study population.

	PwT1DM
Subjects (*N*)	59
Average age (years) at diabetic onset	6.05 ± 3.41 (0.8–13.7)
Time (years) of disease evolution	6.45 ± 3.04 (1–13.2)
Multiple dose insulin therapy (*N*/%)	43/72.9%
Continuous infusion of subcutaneous insulin (insulin pump) therapy (*N*/%)	16/27.1%

**Table 3:** HbA1c levels and variability over time in the PwT1DM group.

HbA1c (%)	*N*	Average	Median	Min–Max
On the date of exploration	59	7.93 ± 1.05	7.50	6.20–14.60
Year prior to exploration	59	7.97 ± 1.25	7.70	6.32–14.10
Historical period of clinical follow-up	58	7.93 ± 0.78	7.88	6.10–12.80

**Table 4:** Retinal thickness and volume reference values for each ETDRS sector, calculated with measurements from healthy controls.

Retinal sector	Percentiles							*N* = 40
	3	10	25	50	75	90	97	Average ± SD
Foveolar thickness (*μ*m)	165	172.5	181	192	205	222	230	194.1 ± 17.9
Total macular volume (mm^3^)	7.07	7.26	7.42	7.60	7.76	8.06	8.20	7.62 ± 0.29
Macular central thickness (*μ*m)	194.3	209	217.2	229	245	255	267.3	230.4 ± 18.7
Superior inner thickness (*μ*m)	272.9	287	295	302	311	318	322.3	301.8 ± 12.9
Temporal inner thickness (*μ*m)	265.3	275	280	289	298	304	310.3	288.3 ± 12
Inferior inner thickness (*μ*m)	269	283	289.2	297	306.7	312.5	321	297.1 ± 12.8
Nasal inner thickness (*μ*m)	282	289	296	304	312	320	328.9	304 ± 11.7
Superior outer thickness (*μ*m)	241.5	247.3	256	263	272	282.7	295	264.8 ± 15.8
Temporal outer thickness (*μ*m)	213.6	233	238	249	256	263.6	273	247.8 ± 13
Inferior outer thickness (*μ*m)	238	241	249	256.5	263	274	288.3	257.6 ± 12.6
Nasal outer thickness (*μ*m)	253.7	263.5	271	280	287	297	307.6	279.8 ± 12.8
Central macular volume (mm^3^)	0.15	0.16	0.17	0.18	0.19	0.20	0.21	0.18 ± 0.01
Superior inner volume (mm^3^)	0.44	0.45	0.46	0.47	0.49	0.50	0.51	0.48 ± 0.08
Temporal inner volume (mm^3^)	0.41	0.43	0.44	0.45	0.47	0.48	0.48	0.45 ± 0.03
Inferior inner volume (mm^3^)	0.42	0.45	0.46	0.47	0.48	0.49	0.5	0.46 ± 0.02
Nasal inner volume (mm^3^)	0.44	0.45	0.46	0.48	0.49	0.50	0.51	0.47 ± 0.02
Superior outer volume (mm^3^)	1.28	1.31	1.35	1.39	1.43	1.49	1.53	1.39 ± 0.07
Temporal outer volume (mm^3^)	1.23	1.24	1.28	1.32	1.36	1.41	1.50	1.32 ± 0.07
Inferior outer volume (mm^3^)	1.26	1.28	1.32	1.36	1.38	1.45	1.52	1.36 ± 0.06
Nasal outer volume (mm^3^)	1.35	1.40	1.44	1.49	1.53	1.57	1.60	1.48 ± 0.06
Total RNFL thickness (*μ*m)	84.5	89	92	97	101	106	112.4	97 ± 6.6
Superior RNFL thickness (*μ*m)	102	107	113	119	126	135	143	120.1 ± 11.0
Inferior RNFL thickness (*μ*m)	98	107	113	120	127	133	138.4	119.9 ± 10.4

**Table 5:** Comparison of retinal thicknesses and volumes between PwT1DM and healthy controls.

	Controls	PwT1DM	*p*
*N* (eyes)	40	110	
Foveolar thickness (*μ*m)	194.1 ± 17.9	194.76 ± 16.91	NS
Total macular volume (mm^3^)	7.62 ± 0.29	7.60 ± 0.27	NS
Macular central thickness (*μ*m)	230.4 ± 18.7	230.03 ± 18.20	NS
Superior inner thickness (*μ*m)	301.8 ± 12.9	301.20 ± 13.31	NS
Temporal inner thickness (*μ*m)	288.31 ± 12.01	287.66 ± 11.88	NS
Inferior inner thickness (*μ*m)	297.1 ± 12.81	296.18 ± 11.97	NS
Nasal inner thickness (*μ*m)	304.27 ± 11.69	303.04 ± 11.36	NS
Superior outer thickness (*μ*m)	264.78 ± 15.8	265.02 ± 16.15	NS
Temporal outer thickness (*μ*m)	247.8 ± 12.97	247.10 ± 13.67	NS
Inferior outer thickness (*μ*m)	257.58 ± 12.62	256.93 ± 12.05	NS
Nasal outer thickness (*μ*m)	279.80 ± 12.81	278.98 ± 12.42	NS
Central macular volume (mm^3^)	0.182 ± 0.014	0.182 ± 0.013	NS
Superior inner volume (mm^3^)	0.481 ± 0.081	0.483 ± 0.098	NS
Temporal inner volume (mm^3^)	0.453 ± 0.035	0.450 ± 0.034	NS
Inferior inner volume (mm^3^)	0.467 ± 0.024	0.466 ± 0.018	NS
Nasal inner volume (mm^3^)	0.478 ± 0.022	0.485 ± 0.092	NS
Superior outer volume (mm^3^)	1.39 ± 0.071	1.39 ± 0.064	NS
Temporal outer volume (mm^3^)	1.32 ± 0.070	1.32 ± 0.073	NS
Inferior outer volume (mm^3^)	1.36 ± 0.061	1.36 ± 0.063	NS
Nasal outer volume (mm^3^)	1.48 ± 0.062	1.48 ± 0.064	NS
Total RNFL thickness (*μ*m)	96.86 ± 6.60	97.37 ± 7.29	NS
Superior RNFL thickness (*μ*m)	120.09 ± 10.97	120.13 ± 11.66	NS
Inferior RNFL thickness (*μ*m)	119.86 ± 10.37	120.08 ± 11.36	NS
Spherical equivalent (D)	0.350 ± 1.67	0.267 ± 1.60	NS

**Table 6:** Comparative study between IDR PwT1DM and control with retinal findings.

Retinal sector	Average ± SD controls	Values Case 1	Percentiles Case 1	Values Case 2	Percentiles Case 2
Foveolar thickness (*μ*m)	194.1 ± 17.9	194	p50–75	206	p75–90
Total macular volume (mm^3^)	7.62 ± 0.29	7.5	p25–50	6.94	<p3
Macular central thickness (*μ*m)	230.4 ± 18.7	224	p25–50	227	p50–75
Superior inner thickness (*μ*m)	301.8 ± 12.9	258	<p3	282	p3–10
Temporal inner thickness (*μ*m)	288.3 ± 12	266	p3–10	271	p3–10
Inferior inner thickness (*μ*m)	297.1 ± 12.8	281	p3–10	279	p3–10
Nasal inner thickness (*μ*m)	304 ± 11.7	281	<p3	283	p3–10
Superior outer thickness (*μ*m)	264.8 ± 15.8	267	p50–75	233	<p3
Temporal outer thickness (*μ*m)	247.8 ± 13	241	p25–50	223	p3–10
Inferior outer thickness (*μ*m)	257.6 ± 12.6	260	p50–75	233	<p3
Nasal outer thickness (*μ*m)	279.8 ± 12.8	281	p50–75	255	p3–10
Central macular volume (mm^3^)	0.18 ± 0.01	0.18	p50	0.18	p50
Superior inner volume (mm^3^)	0.48 ± 0.08	0.45	p10	0.44	p3
Temporal inner volume (mm^3^)	0.45 ± 0.03	0.42	p3–10	0.42	p3–10
Inferior inner volume (mm^3^)	0.46 ± 0.02	0.44	p3–10	0.44	p3–10
Nasal inner volume (mm^3^)	0.47 ± 0.02	0.45	p3–10	0.44	p3
Superior outer volume (mm^3^)	1.39 ± 0.07	1.42	p50–75	1.24	<p3
Temporal outer volume (mm^3^)	1.32 ± 0.07	1.29	p25–50	1.18	<p3
Inferior outer volume (mm^3^)	1.36 ± 0.06	1.38	p75	1.23	<p3
Nasal outer volume (mm^3^)	1.48 ± 0.06	1.49	p50	1.35	p3
Total RNFL thickness (*μ*m)	97 ± 6.6	107	p90–97	91	p10–25
Superior RNFL thickness (*μ*m)	120.1 ± 11.0	135	p90	97	<p3
Inferior RNFL thickness (*μ*m)	119.9 ± 10.4	140	>p97	117	p25–50

## References

[1] Cheung N., Mitchell P., Wong T. Y. (2010). Diabetic retinopathy. *The Lancet*.

[2] Yau J. W. Y., Rogers S. L., Kawasaki R. (2012). Global prevalence and major risk factors of diabetic retinopathy. *Diabetes Care*.

[3] Cunha-Vaz J., Ribeiro L., Lobo C. (2014). Phenotypes and biomarkers of diabetic retinopathy. *Progress in Retinal and Eye Research*.

[4] Antonetti D. A., Klein R., Gardner T. W. (2012). Diabetic retinopathy. *The New England Journal of Medicine*.

[5] Kur J., Newman E. A., Chan-Ling T. (2012). Cellular and physiological mechanisms underlying blood flow regulation in the retina and choroid in health and disease. *Progress in Retinal and Eye Research*.

[6] Bearse M. A., Adams A. J., Han Y. (2006). A multifocal electroretinogram model predicting the development of diabetic retinopathy. *Progress in Retinal and Eye Research*.

[7] Bearse M. A., Ozawa G. Y. (2014). Multifocal electroretinography in diabetic retinopathy and diabetic macular edema. *Current Diabetes Reports*.

[8] Oshitari T., Hanawa K., Adachi-Usami E. (2009). Changes of macular and RNFL thicknesses measured by stratus OCT in patients with early stage diabetes. *Eye*.

[9] Hovind P., Tarnow L., Rossing K. (2003). Decreasing incidence of severe diabetic microangiopathy in type 1 diabetes. *Diabetes Care*.

[10] Kytö J. P., Harjutsalo V., Forsblom C. (2011). Decline in the cumulative incidence of severe diabetic retinopathy in patients with type 1 diabetes. *Diabetes Care*.

[11] Klein R., Klein B. E., Moss S. E., Davis M. D., DeMets D. L. (1984). The Wisconsin epidemiologic study of diabetic retinopathy. II. Prevalence and risk of diabetic retinopathy when age at diagnosis is less than 30 years. *Archives of Ophthalmology*.

[12] Svensson M., Eriksson J. W., Dahlquist G. (2004). Early glycemic control, age at onset, and development of microvascular complications in childhood-onset type 1 diabetes: a population-based study in northern Sweden. *Diabetes Care*.

[13] Altemir I., Pueyo V., Elía N., Polo V., Larrosa J. M., Oros D. (2013). Reproducibility of optical coherence tomography measurements in children. *American Journal of Ophthalmology*.

[14] Chung H. K., Han Y. K., Oh S., Kim S. H. (2016). Comparison of optical coherence tomography measurement reproducibility between children and adults. *PLoS One*.

[15] Grover S., Murthy R. K., Brar V. S., Chalam K. V. (2009). Normative data for macular thickness by high-definition spectral-domain optical coherence tomography (Spectralis). *American Journal of Ophthalmology*.

[16] Leung C. K., Cheung C. Y., Weinreb R. N. (2008). Comparison of macular thickness measurements between time domain and spectral domain optical coherence tomography. *Investigative Ophthalmology & Visual Science*.

[17] Pierro L., Gagliardi M., Iuliano L., Ambrosi A., Bandello F. (2012). Retinal nerve fiber layer thickness reproducibility using seven different OCT instruments. *Investigative Ophthalmology & Visual Science*.

[18] Pierro L., Giatsidis S. M., Mantovani E., Gagliardi M. (2010). Macular thickness interoperator and intraoperator reproducibility in healthy eyes using 7 optical coherence tomography instruments. *American Journal of Ophthalmology*.

[19] Buchser N. M., Wollstein G., Ishikawa H. (2012). Comparison of retinal nerve fiber layer thickness measurement bias and imprecision across three spectral-domain optical coherence tomography devices. *Investigative Ophthalmology & Visual Science*.

[20] Goebel W., Kretzchmar-Gross T. (2002). Retinal thickness in diabetic retinopathy: a study using optical coherence tomography (OCT). *Retina*.

[21] Hee M. R., Puliafito C. A., Wong C. (1995). Quantitative assessment of macular edema with optical coherence tomography. *Archives of Ophthalmology*.

[22] Lattanzio R., Brancato R., Pierro L. (2002). Macular thickness measured by optical coherence tomography (OCT) in diabetic patients. *European Journal of Ophthalmology*.

[23] Massin P., Girach A., Erginay A., Gaudric A. (2006). Optical coherence tomography: a key to the future management of patients with diabetic macular oedema. *Acta Ophthalmologica Scandinavica*.

[24] Virgili G., Menchini F., Casazza G. (2015). Optical coherence tomography (OCT) for detection of macular oedema in patients with diabetic retinopathy. *Cochrane Database of Systematic Reviews*.

[25] Verma A., Rani P. K., Raman R. (2009). Is neuronal dysfunction an early sign of diabetic retinopathy? Microperimetry and spectral domain optical coherence tomography (SD-OCT) study in individuals with diabetes, but no diabetic retinopathy. *Eye*.

[26] Elhabashy S. A., Elbarbary N. S., Nageb K. M., Mohammed M. M. (2015). Can optical coherence tomography predict early retinal microvascular pathology in type 1 diabetic adolescents without minimal diabetic retinopathy? A single-centre study. *Journal of Pediatric Endocrinology and Metabolism*.

[27] Sayin N., Kara N., Pirhan D. (2014). Evaluation of subfoveal choroidal thickness in children with type 1 diabetes mellitus: an EDI-OCT study. *Seminars in Ophthalmology*.

[28] Koleva-Georgieva D. N., Sivkova N. P. (2010). Optical coherence tomography for the detection of early macular edema in diabetic patients with retinopathy. *Folia Medica*.

[29] El-Fayoumi D., Badr Eldine N. M., Esmael A. F., Ghalwash D., Soliman H. M. (2016). Retinal nerve fiber layer and ganglion cell complex thicknesses are reduced in children with type 1 diabetes with no evidence of vascular retinopathy. *Investigative Ophthalmology & Visual Science*.

[30] The Diabetes Control and Complications Trial Research Group (1993). The effect of intensive treatment of diabetes on the development and progression of long-term complications in insulin-dependent diabetes mellitus. *The New England Journal of Medicine*.

[31] Klein R., Klein B. E., Moss S. E. (1990). The Wisconsin epidemiologic study of diabetic retinopathy: an update. *Australian and New Zealand Journal of Ophthalmology*.

[32] Bonney M., Hing S. J., Fung A. T. (1995). Development and progression of diabetic retinopathy: adolescents at risk. *Diabetic Medicine*.

[33] Stephenson J., Fuller J. H., EUROBIAB IDDM Complications Study Group (1994). Microvascular and acute complications in IDDM patients: the EURODIAB IDDM Complications Study. *Diabetologia*.

[34] Geloneck M. M., Forbes B. J., Shaffer J., Ying G., Binenbaum G. (2015). Ocular complications in children with diabetes mellitus. *Ophthalmology*.

[35] Downie E., Craig M. E., Hing S., Cusumano J., Chan A. K. F., Donaghue K. C. (2011). Continued reduction in the prevalence of retinopathy in adolescents with type 1 diabetes: role of insulin therapy and glycemic control. *Diabetes Care*.

[36] Cho Y. H., Craig M. E., Hing S. (2011). Microvascular complications assessment in adolescents with 2- to 5-yr duration of type 1 diabetes from 1990 to 2006. *Pediatric Diabetes*.

[37] Hermann J. M., Hammes H.-P., Rami-Merhar B. (2014). HbA_1c_ variability as an independent risk factor for diabetic retinopathy in type 1 diabetes: A German/Austrian multicenter analysis on 35,891 patients. *PLoS One*.

[38] Hsu C.-R., Chen Y.-T., Sheu W. H.-H. (2015). Glycemic variability and diabetes retinopathy: a missing link. *Journal of Diabetes and its Complications*.

[39] Kilpatrick E. S., Rigby A. S., Atkin S. L. (2008). A1C variability and the risk of microvascular complications in type 1 diabetes: data from the Diabetes Control and Complications Trial. *Diabetes Care*.

[40] Škrha J., Šoupal J., Škrha J., Prázný M. (2016). Glucose variability, HbA1c and microvascular complications. *Reviews in Endocrine and Metabolic Disorders*.

[41] Suh S., Kim J. H. (2015). Glycemic variability: how do we measure it and why is it important?. *Diabetes & Metabolism Journal*.

[42] Fullerton B., Jeitler K., Seitz M., Horvath K., Berghold A., Siebenhofer A. (2014). Intensive glucose control versus conventional glucose control for type 1 diabetes mellitus. *Cochrane Database of Systematic Reviews*.

[43] Salardi S., Porta M., Maltoni G. (2016). Ketoacidosis at diagnosis in childhood-onset diabetes and the risk of retinopathy 20 years later. *Journal of Diabetes and its Complications*.

[44] Dirani M., Xie J., Fenwick E. (2011). Are obesity and anthropometry risk factors for diabetic retinopathy?: The diabetes management project. *Investigative Ophthalmology & Visual Science*.

